# AggLb Is the Largest Cell-Aggregation Factor from *Lactobacillus paracasei* Subsp. *paracasei* BGNJ1-64, Functions in Collagen Adhesion, and Pathogen Exclusion *In Vitro*

**DOI:** 10.1371/journal.pone.0126387

**Published:** 2015-05-08

**Authors:** Marija Miljkovic, Ivana Strahinic, Maja Tolinacki, Milica Zivkovic, Snezana Kojic, Natasa Golic, Milan Kojic

**Affiliations:** 1 Laboratory for Molecular Microbiology, Institute of Molecular Genetics and Genetic Engineering, University of Belgrade, Vojvode Stepe 444/a, P.O. Box 23, 11010, Belgrade, Serbia; 2 Laboratory for Molecular Biology, Institute of Molecular Genetics and Genetic Engineering, University of Belgrade, Vojvode Stepe 444/a, P.O. Box 23, 11010, Belgrade, Serbia; Catalan Institute for Water Research (ICRA), SPAIN

## Abstract

Eleven *Lactobacillus* strains with strong aggregation abilities were selected from a laboratory collection. In two of the strains, genes associated with aggregation capability were plasmid located and found to strongly correlate with collagen binding. The gene encoding the auto-aggregation-promoting protein (AggLb) of *Lactobacillus paracasei* subsp. *paracasei* BGNJ1-64 was cloned using a novel, wide-range-host shuttle cloning vector, pAZILSJ. The clone pALb35, containing a 11377-bp DNA fragment, was selected from the SacI plasmid library for its ability to provide carriers with the aggregation phenotype. The complete fragment was sequenced and four potential ORFs were detected, including the *aggLb* gene and three surrounding transposase genes. AggLb is the largest known cell-surface protein in lactobacilli, consisting of 2998 aa (318,611 Da). AggLb belongs to the collagen-binding superfamily and its C-terminal region contains 20 successive repeats that are identical even at the nucleotide level. Deletion of *aggLb* causes a loss of the capacity to form cell aggregates, whereas overexpression increases cellular aggregation, hydrophobicity and collagen-binding potential. PCR screening performed with three sets of primers based on the *aggLb* gene of BGNJ1-64 enabled detection of the same type of *aggLb* gene in five of eleven selected aggregation-positive *Lactobacillus* strains. Heterologous expression of *aggLb* confirmed the crucial role of the AggLb protein in cell aggregation and specific collagen binding, indicating that AggLb has a useful probiotic function in effective colonization of host tissue and prevention of pathogen colonization.

## Introduction

Due to their long history of safe use in food fermentation and preservation, lactobacilli currently carry the ‘Qualified Presumption of Safety’ (QPS) status [1]. Particularly, lactobacilli have attracted attention as probiotic due to beneficial effects on human and animal hosts. According to the FAO/WHO (2006) guidelines for the evaluation of probiotics for human food applications [2], one of the important criteria for probiotic selection is the capability to adhere to the host’s intestinal epithelium. It is believed that adherence ability is important for successful colonization and achievement of favorable effects over a longer period of time. The ability of probiotic bacteria to adhere to epithelial surfaces has been extensively analyzed [3, 4]. The precise mechanisms that affect crosstalk between the microbe and host remain unclear, although there is growing evidence that adherence depends on bacterial cell-surface composition. Probiotic microorganisms express cell-surface adhesins that mediate microbial adhesion to the extracellular matrix (ECM) components of host tissue such as mucin, fibronectin, collagen, laminin or fibrinogen [5]. For example, a 43-kDa collagen-binding S-layer protein has been identified in *Lactobacillus crispatus* [6], and Lorca *et al*. [7] showed that a 15-kDa protein from *Lactobacillus acidophilus* CRL 639 binds fibronectin while two proteins of 45- and 58-kDa interact with collagen. On the other hand, various human pathogenic bacteria also exhibit specific adhesiveness to collagenous proteins [8, 9]. This interaction is critical in early-phase infection, and thus is strongly related to the virulence of the pathogen. Through the action of cell-surface adhesins, pathogens successfully interact with proteins of the ECM, preserving peristalsis and enabling colonization of the tissue and infection [9]. An example is the collagen-binding protein that enables *Staphylococcus aureus* cells to adhere to cartilage *in vitro* [10] and which has been described as a major virulence factor.

Cellular aggregation is defined as ability of cells to form precipitates. Auto-aggregation involves cells of the same bacterial strain, while genetically distant cells co-aggregate [11]. The aggregation phenomenon has primarily been connected with a high frequency of genetic exchange through the easily achieved communication between cells [12]. In lactobacilli, the two types of proteins responsible for manifestation of the aggregation phenomenon are the soluble proteins and the cell-surface proteins [13, 14]. Co-aggregation promoting factors from lactobacilli differ in molecular weight and primary structure [11, 15, 16, 17]. The smallest described protein (2 kDa) mediates self-aggregation and was found in the vaginal isolate *Lactobacillus gasseri* 2459. This protein likely acts as a pheromone-like factor that induces expression of surface proteins that are critical for adhesion [13]. On the other hand, a very large protein (molecular mass > 200 kDa) directly involved in aggregation was described in *Lactobacillus paracasei* subsp. *paracasei* BGSJ2-8 [18]. In addition to the proteinaceous component, various ions are linked to aggregation in certain lactobacilli strains that have been isolated from cheese [14, 18].

Cell-surface proteins are likely linked to the efficient interaction of probiotic bacteria with ECM proteins. It is possible that this interaction helps the formation of a barrier that inhibits the adherence of pathogens to the intestinal mucosa, thereby preventing pathogenic colonization and ultimately assisting in the removal of pathogens from the intestinal environment. Indeed, the presence of common receptors in different types of bacteria, e.g., *Lactobacillus* and select pathogens, could enable the exclusion of pathogens from the gastrointestinal (GI) and urogenital (UG) tracts by direct competition for binding sites on epithelial cell-surface proteins [19].

This study aimed to investigate the relationship between auto-aggregation, binding to different surfaces and pathogen exclusion *in vitro*, in selected *Lactobacillus* strains from a laboratory collection. The novel *aggLb* gene coding for the auto-aggregation-promoting protein of *Lb*. *paracasei* subsp. *paracasei* BGNJ1-64 was cloned and functionally analyzed. Given the fact that the biological function of the aggregation phenomenon has remained poorly characterized, deciphering the structural characteristics and binding specificity of AggLb is important for improving our understanding of the mechanisms involved in probiotic-host interactions.

## Materials and Methods

### Bacterial strains and growth conditions

The bacterial strains, their derivatives, and the plasmids used in this study are listed in Table 1. *Lactobacillus paracasei* was grown in de Man, Rogosa, and Sharpe (MRS) (Merck GmbH, Darmstadt, Germany) medium at 30°C. *Lactococcus lactis* subsp. *lactis* and *Enterococcus faecalis* were grown at 30°C in M17 medium (Merck GmbH, Darmstadt, Germany) supplemented with 0.5% glucose (GM17). *Staphylococcus aureus*, *Pseudomonas aeruginosa* PAO1 and *Escherichia coli* DH5α and O157:H7 were grown in Luria-Bertani medium (LB) at 37°C with aeration. Erythromycin was added for a final concentration of 10 μg/ml or 300 μg/ml for lactic acid bacteria (LAB) and *E*. *coli*, respectively. Ampicillin was added for a final concentration of 100 μg/ml for *E*. *coli*. When necessary, 5-bromo-4-chloro-3-indolyl-β-D-galactoside (X-Gal) (Fermentas, Vilnius, Lithuania) was added to LB plates for a final concentration of 40 μg/ml for blue/white color selection of colonies.

**Table 1:** List of bacterial strains and plasmids used in the study.

	General characteristics	Source or reference
Strains		
*Lactobacillus paracasei* subsp. *paracasei*		
BGSJ2-8	Agg^+^, Prt^+^, Bac^+^	[18]
BGSJ2-81	Agg^-^, Prt^+^, Bac^+^	[18]
BGAR75	Agg^+^, Prt^+^, Bac^+^	[14]
BGGR2-68	Agg^+^	[14]
BGGR2-82	Agg^+^	[14]
BGDP1-84	Agg^+^	[14]
BGDP9-38	Agg^+^, Prt^+^	This work
BGNJ1-3	Agg^+^, Prt^+^	[14]
BGNJ1-61	Agg^+^, Prt^+^, Bac^+^	[14]
BGNJ1-64	Agg^+^ Bac^+^	This work
BGNJ1-641	Agg^-^	This work
BGNJ1-70	Agg^+^, Bac^+^	[14]
BGZLS30-6	Agg^+^	[20]
*Lactococcus lactis* subsp. *lactis*		
BGKP1-20	Agg^-^	[21]
BGKP1-20/pALb35	Agg^+^, Em^r^	This work
*Enterococcus faecalis*		
BGZLS10-27	Agg^-^	[22]
BGZLS10-27/pALb35	Agg^+^, Em^r^	This work
*Escherichia coli*		
DH5α	F^-^ Φ80d*lacZ*ΔM15 Δ(*lacZYA*-*argF*) *U169 recA1 endA1 hsdR17* (r_K_^-^ m_K_^-^) *supE44 thi-1 gyrA relA1*	[23]
O157:H7		ATCC35150
*Staphylococcus aureus*		ATCC25923
*Pseudomonas aeruginosa*		
PAO1		Laboratory collection
Plasmids		
pGEM-T Easy Vector	3015 bp, Amp^r^, bacterial, nonviral, transient, constitutive, high expression level	Promega
pAZIL	Em^r^, shuttle cloning vector	[21]
pAZILSJ cloning vector	8589 bp, derivative of pAZIL vector carrying origin of replication and *repB* gene from plasmid pSJ2-8	This work
pNJ1	~40 kb, natural plasmid of BGNJ1-64 carrying *aggLb* gene	This work
pALb35	pAZILSJ derivative carrying 11377 bp SacI fragment of pNJ1 plasmid	This work

Amp^r^ **-** resistance to ampicillin; Em^r^ **-** resistance to erythromycin; Bac^+^ **-** bacteriocin producer; Prt^+^ **-** proteolytically active; Agg^+^ **-** aggregation-positive; Agg^—^ aggregation-negative.

### Recombinant DNA techniques

The non-aggregating derivatives *L*. *lactis* subsp. *lactis* BGKP1-20 and *E*. *faecalis* BGZLS10-27 were used for heterologous expression of aggregation factor. Electrocompetent BGKP1-20 and BGZLS10-27 cells were prepared as described by Holo and Nes [24]. With the exception of *E*. *coli* DH5α, which were transformed by heat shock [23], transformations were performed by electroporation using an Eppendorf Electroporator (Eppendorf, Hamburg, Germany). Agar plates with the appropriate antibiotic were used for the selection of transformants.

Plasmid DNA from *E*. *coli* DH5α was isolated using a QIAprep Spin Miniprep kit (Qiagen GmBH, Hilden, Germany). The procedure described by O’Sullivan and Klaenhammer was applied for plasmid isolation from lactococci and lactobacilli [25]. Curing experiments were performed as described previously [26]. For cloning experiments, plasmid DNA from lactobacilli was isolated according to methods from Anderson and Mc Kay [27] and separated using CsCl-gradient ultra-centrifugation [28]. Total DNA from pure cultures was extracted by the method described by Hopwood *et al*. [29]. The DNA fragments from agarose gels were purified using a QIAquick Gel Extraction kit (Qiagen GmBH, Hilden, Germany). Digestion with restriction enzymes HindIII, PstI, SacI, EcoRI, SalI, and BamHI was conducted according to the supplier's instructions (Fermentas, Vilnius, Lithuania). The sequences and localization of the primers used in this study are shown in S1 Table. PCR was carried out in a 2720 Thermal Cycler (Applied Biosystems, Foster City, CA) in a final volume of 50 μl with 1.25 U of KAPA *Taq* DNA polymerase (Kapa Biosystems, Woburn, USA), 20 ng of genomic DNA, 200 μmol dNTPs each (Invitrogen, Cergy-Pontoise, France) and 25 μmol of each primer. After an initial denaturation step of 5 min at 94°C, the mixture was subjected to 30 cycles of three steps (denaturation at 95°C, annealing at 55°C, and polymerization at 72°C, each for 30 s). A final extension was performed for 7 min at 72°C. PCR products were checked on a 1% (wt/vol) agarose gel that was run at a constant voltage. The ready-to-use GeneRuler 1 kb Plus DNA Ladder (Fermentas) was used as a molecular-weight marker.

Sub-cloning of HindIII, SspI and HincII fragments and deletion experiments with exonuclease III (ExoIII)/S1 enabled sequencing of the repeat-containing *aggLb* gene region. Series of nested deletions of the pALb35 plasmid were obtained by digestion with EcoRV and SacI, which enabled unidirectional ExoIII activity. The resulting DNA was progressively shortened from the EcoRV end by approximately 100 nucleotides per minute. SacI/EcoRV-predigested pALb35 DNA was digested for 5, 10, 15, 20, 25, 30, 35 and 40 min with ExoIII at 30°C in the presence of 100 mM NaCl. Resulting deletions ranged from 500 to 4000 bp. The ends of deletion fragments were blunted by S1, ligated and transformed into *E*. *coli* DH5α. Plasmid DNA isolated from transformants was screened by digestion with EcoRI and SalI restriction enzymes. Selected deletion mutants were sequenced.

Southern blot hybridization was performed with the DIG DNA Labeling and Detection Kit (Roche Applied Science, Mannheim, Germany) following the instructions of the manufacturer.

### Construction of pAZILSJ vector

The vector pAZILSJ, a LAB *E*. *coli* shuttle-cloning vector, is a derivative of the pAZIL vector [21]. The vector pAZILSJ was constructed to enable molecular analysis of cloned fragments in lactobacilli. The 1480-bp BclI fragment, carrying the origin of replication and *repB1* gene from the lactobacilli plasmid pSJ2-8 [30], was inserted into the BclI site of the pAZIL vector and selected in *E*. *coli* DH5α (on erythromycin 300 μg/ml). The presence of *ori* and *repB1* in the construct was confirmed by PCR amplification with BclIF and BclIR primers (S1 Table) and sequencing. The ability of pAZILSJ to replicate in different lactobacilli strains was tested.

### Construction of BGNJ1-64 and BGSJ2-8 plasmid libraries

To enable the expression of the *Lactobacillus agg* gene(s) in different *Lactococcus* and *Lactobacillus* strains, an improved derivative of the pAZIL vector was constructed. The pAZILSJ was obtained after insertion of the 1.4 kb BclI fragment from the pSJ2-8 plasmid into pAZIL BclI fragment carrying the origin of replication and the *repB1* gene. For preparation of plasmid libraries, plasmid DNA from BGNJ1-64 and BGSJ2-8 was purified by CsCl gradient centrifugation and digested with different restriction enzymes (EcoRI, PstI, SacI, BamHI and SalI). Obtained DNA fragments were cloned into the pAZILSJ vector predigested with the same restriction enzymes. The plasmid libraries were first screened in *E*. *coli* DH5α (blue-white selection on LA plates containing erythromycin and X-gal). All plasmid derivatives carrying different fragments were transferred into non-aggregating cells. Heterologous lactococcal derivative BGKP1-20 was used because the original *Lactobacillus* derivative showed an extremely low efficiency of transformation. Only the construct pALb35, carrying SacI fragment originating from the plasmid library of BGNJ1-64, restored the auto-aggregation ability of BGKP1-20.

### Sequencing and sequence analysis

Both DNA strands were sequenced by Macrogen sequencing service (Amsterdam, The Netherlands). Sequence annotation and database searches for sequence similarities were completed using BLAST (National Center for Biotechnology Information) [31]. The DNA Strider program was used for ORF prediction. Motif Scan (http://myhits.isb-sib.ch/cgi-bin/motif_scan), DAS Transmembrane Domain [32], and Superfamily 1.75 (http://supfam.org/) software were used for *in silico* analysis of the AggLb protein. The DNA sequence of the SacI/SacI fragment (11377 bp) carrying the *aggLb* gene from the pNJ1 plasmid was submitted to the EMBL GenBank under accession number HG008907.2.

### Visual aggregation assay

From the 1000 *Lactobacillus* strains from laboratory collection, the strains that exhibited aggregation ability were screened by a visual assay. Aggregation was scored as positive when clearly visible sand-like particles formed by aggregated cells gravitated to the bottom of the tube, formed a precipitate and left a clear supernatant.

### Auto-aggregation and co-aggregation assays

The auto-aggregation and co-aggregation abilities of the selected lactobacillus strains were tested according to Garcia *et al*. [33] with minor modifications. Briefly, cells from overnight culture were harvested by centrifugation (5000 × g, 10 min, 4°C), washed twice with phosphate-buffered saline (PBS) pH 7.1 (10 mM Na_2_HPO_4_, 1 mM KH_2_PO_4_, 140 mM NaCl, 3 mM KCl) and resuspended in the same buffer. The mixture was vortexed and incubated at 30°C for 5 h. Absorbance (OD_600_) was measured at different time points. The percentage of auto-aggregation was determined using the equation: [1 − (A_t_/A_0_) × 100], where A_t_ represents the absorbance at different time points (1, 2, 3, 4 and 5 h) and A_0_ is absorbance at time 0. Lactobacilli strains were classified as strongly autoaggregating (% of autoaggregation after 1 h > 30), weakly autoaggregating (30 < % of autoaggregation after 1 h > 10) or non- autoaggregating (% of autoaggregation after 1 h < 10). For the analysis of co-aggregation between the selected lactobacillus and pathogen strains, bacterial suspensions were prepared as described above and mixed in a ratio of 1:1. The degree of co-aggregation was expressed as [(((A_path_ + A_lb_)/2 –(A_mix_))/((A_path_ + A_lb_)/2)) × 100], where A_path_ and A_lb_ represent the absorbance in control samples containing the pathogen or the lactobacilli strain and A_mix_ represents the absorbance of the mixture at different time points.

### Biofilm assay

The ability of tested lactobacillus and pathogen strains to form biofilms was assayed in microtiter plates as previously described by Peter *et al*. [34]. *P*. *aeruginosa* PAO1 and *E*. *coli* DH5α were used as positive and negative control strains, respectively.

### Collagen-binding assay

The wells of Maxisorb plates (Nunc, Roskilde, Denmark) were coated with type I collagen (from rat tail, BD Bioscience, New Jersey, United States) (100 μg/ml) for 16 h at 4°C. After immobilization, wells were washed with PBS and blocked with 2% BSA in PBS. Upon removal of the BSA solution and washing the wells with PBS, the test cultures (100 μl, 10^8^ CFU/ml) were added and plates were incubated on an orbital platform shaker for 2 h at 37°C. Non-adherent cells were removed by washing the wells three times with 200 μl of PBS. The adhered cells were fixed at 60°C for 20 min and stained with crystal violet (100 μl/well, 0.1% solution) for 45 min. Wells were subsequently washed three times with PBS to remove the excess stain. The stain bound to the cells was dissolved in 100 μl of citrate buffer (pH 4.3). The absorbance was measured at 570 nm after 45 min using the microtiter plate reader. Results were expressed as the mean of six replicates normalized to the negative control, according to Vesterlund *et al*. [10]. Lactobacilli were classified as strongly adherent (A_570_ > 0.3), weakly adherent (0.1 < A_570_ > 0.3) or non-adherent (A_570_ < 0.1). For inhibition experiments, bacterial suspensions (100 μl, 10^8^ CFU/ml) of the *Lactobacillus* strains with collagen-binding ability and control strains were pre-incubated without or with an equal volume of heparin sodium salt from porcine intestinal mucosa (Sigma, Germany) at a final concentration of 1 mg/ml for 1 h at 30°C. The test cultures were added to microtiter plate wells pre-coated with collagen and incubated on an orbital platform shaker for 2 h at 37°C. Collagen binding was assayed as described above and the average of six absorbance values for each strain was compared with that of the same non-treated strains. The coherence of the results was confirmed in three independent experiments (one experiment per time, in six wells per strain). Data are presented as mean values obtained from one representative experiment. The significance was determined by Student's *t*-test.

### Competitive exclusion assay

The competitive exclusion assay of selected *Lactobacillus* strains against pathogen (*S*. *aureus* ATCC25923 and *E*. *coli* O157:H7 as representatives of Gram positive and Gram negative bacteria, respectively) was performed as reported by Yadav *et al*. [5] with minor modifications. The wells of Maxisorb plates (Nunc, Roskilde, Denmark) were coated with type I collagen (from rat tail, BD Bioscience) (100 μg/ml) and subsequently incubated overnight at 4°C. Collagen solution was removed and wells were washed with PBS. Fresh bacterial culture (100 μl, 10^8^ CFU/ml) was added and plates were incubated for 3 h at 37°C. After washing and removal of unbound bacteria, 100 μl of *S*. *aureus* ATCC25923 or *E*. *coli* O157:H7 culture suspension (10^8^ CFU/ml) in PBS was added, and the plates were incubated at 37°C for an additional 3 h. *Lactobacillus* and pathogen bacteria that bound to collagen were removed by 0.1% Triton X-100 solution for 30 min and plated on appropriate agar plates. After overnight incubation, colonies were counted. The inhibition of adhesion was expressed as the difference between adhesion of pathogen in the absence and presence of tested *Lactobacillus* strains.

### Microscopic analysis

Bacterial cells were stained with DNA fluorescent dye hexidium iodide (HI; excitation/emission at 480 nm/625 nm). Bacterial cultures (2 ml; 10^8^ CFU/ml) were harvested and washed twice with PBS. To characterize the proteinaceous nature of the factor(s) involved in aggregation, bacterial cells were resuspended in PBS containing proteinase K (1 mg/ml) (Merck, Darmstadt, Germany) and incubated for 1 h at 37°C prior staining. Cells were then incubated with 0.03 μmol HI for 15 min at room temperature under constant rotation in the dark. After washing with PBS, stained cells were added to glass bottom dishes and assayed by confocal laser scanning microscopy using a Leica SP8 system (Leica Microsystems GmbH, Wetzlar, Germany). Images were acquired and processed with the Leica LAS AF software.

### Microbial adhesion to solvents (MATS) assay

The MATS assay was performed according to the method described previously [35]. Overnight cultures were harvested by centrifugation at 5000 × g for 10 min, washed twice with 0.1 M KNO_3_ (pH 6.2), and resuspended in the same buffer to an optical density of 0.4 at 400 nm (A_0_). 1.2 ml of cell suspension and 0.2 ml of solvent were pre-incubated for 10 min at room temperature. The suspension was vortexed for 1 min and the two phases were allowed to separate for 15 min. The aqueous phase was removed and optical density at 400 nm (A_t_) was measured. The bacterial affinities to different solvents were estimated as follows: % of bacterial cells adhered to solvent = [1- (A_t_/A_0_) × 100].

## Results

### Screening of aggregation ability among natural isolates of lactobacilli

**Fig 1:**
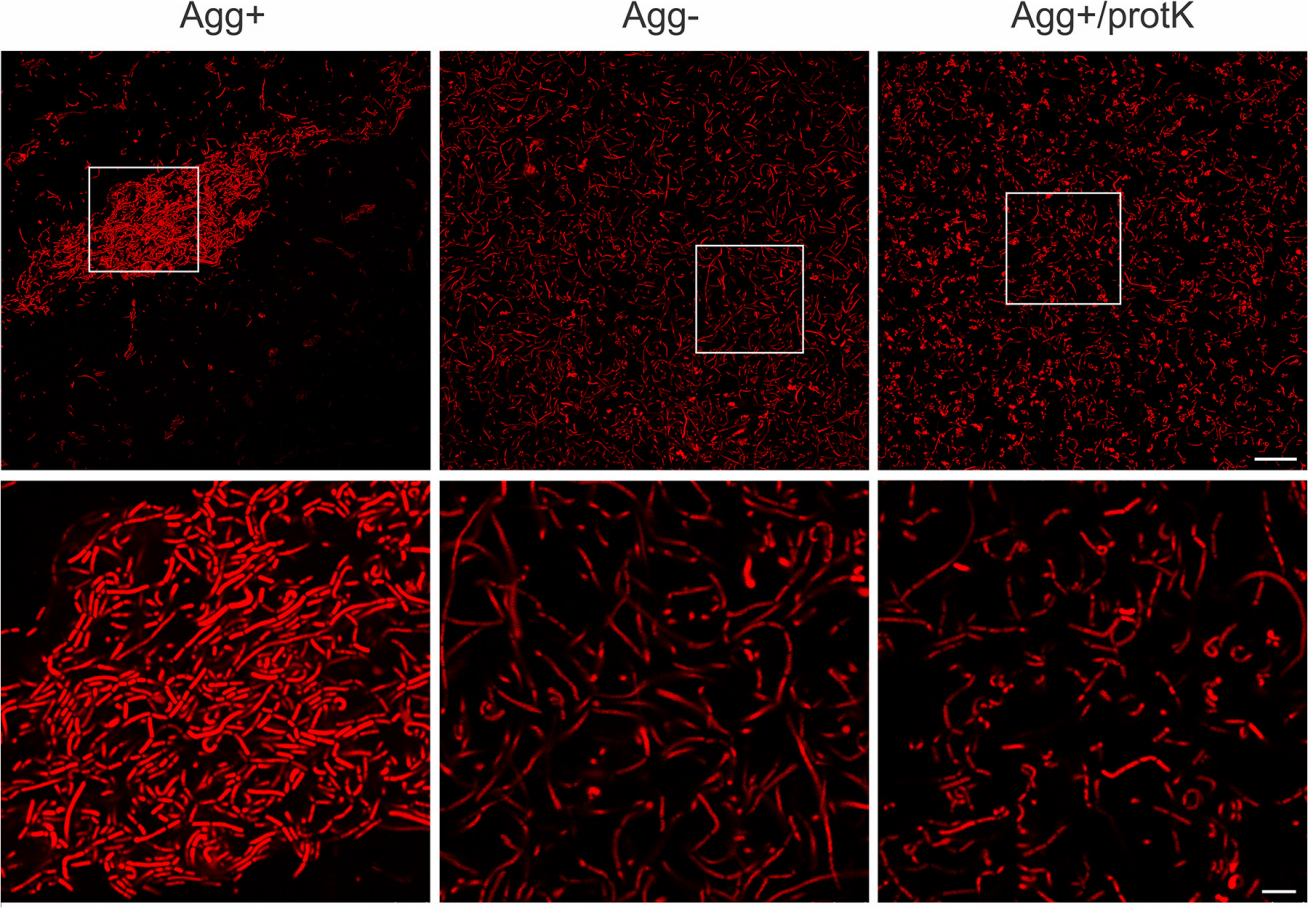
Images of *Lb*. *paracasei* subsp. *paracasei* BGNJ1-64 obtained by confocal microscopy. Agg^+—^microscopic analysis of cells of auto-aggregation positive strain *Lb*. *paracasei* subsp. *paracasei* BGNJ1-64; Agg^-^—cells of the aggregation-deficient variant of this strain, not able to form multicellular structures; Agg^+^/protK—cells of strain BGNJ1-64 treated with proteinase K with loss of ability to form multicellular structures. Higher magnification images are presented on lower panel. Bacteria were stained with hexidium iodide fluorescent dye (excitation maximum of 480 nm and emission maximum of 625 nm). Scale bars are 25 μm for the upper and 5 μm for the lower panel.

The 11 *Lactobacillus* strains showing an auto-aggregation phenotype were selected by the aggregation visual assay (Table 1). All selected strains formed snowflake-like aggregates that became visible after a few seconds of culture vortexing; within one minute, more than 80% of cells precipitated to the bottom of the tube (S1 Fig), giving a completely transparent supernatant. Among them, *Lb*. *paracasei* subsp. *paracasei* BGNJ1-64 was chosen for confocal microscopic observation of multicellular aggregates because it exhibited the strongest auto-aggregation phenotype. As shown in Fig 1, (Agg^+^) fluorescently stained cells were able to form multicellular clumps. As expected, multicellular structures were not observed for aggregation-deficient variants (Fig 1, Agg^-^). Because exhibition of the auto-aggregation phenotype should be related to the surface proteins, the susceptibility of the aggregation determinants to protease inactivation was tested by incubation of bacterial cells with proteinase K. This treatment prevented formation of aggregates (Fig 1, Agg^+^/protK), confirming the proteinaceous nature of aggregation factor.

### Auto-aggregation, co-aggregation and biofilm formation of selected lactobacilli

The visually observed auto-aggregation ability was further confirmed by absorbance (OD_600_) measurement of cultures during 5 h of sedimentation by gravity at 30°C. Tested lactobacilli showed a wide range (38.26–81.34%) of auto-aggregation degrees (S2 Table).

The co-aggregation ability was tested using BGNJ1-64 and *S*. *aureus* ATCC25923. Visible co-aggregates were not detected either by spectrophotometric assay or by confocal microscopy. The lack of aggregates could be attributed to the competition between auto- and co-aggregation processes or insufficient time allowed for interaction between the cells of the *Lactobacillus* and pathogen strains.

Among tested lactobacilli, only BGGR2-82 demonstrated a weak ability to form biofilms on the plastic surface of tissue-culture plates. This result clearly indicates that the aggregation phenomenon present in lactobacilli is most likely not linked to biofilm formation. Moreover, pathogenic *S*. *aureus* ATCC25923 and *E*. *coli* 0157:H7 did not show ability to form biofilms.

### Localization of genes correlated with auto-aggregation ability

Because selected Agg^+^ strains harbor autochthonous plasmids (S2 Fig), the plasmid location of genes responsible for aggregation was determined. To substantiate the relationship between the presence of plasmids in cells and the auto-aggregation phenotype, a plasmid curing experiment was performed with all selected lactobacilli. Agg^-^ derivatives were obtained for BGNJ1-64 and BGSJ2-8, which had different plasmid profiles compared to the parental strains. Obtained results strongly indicate that the gene(s) determining the presence of auto-aggregation in these two strains are located on the plasmid.

### Collagen-binding properties of selected lactobacilli

**Fig 2:**
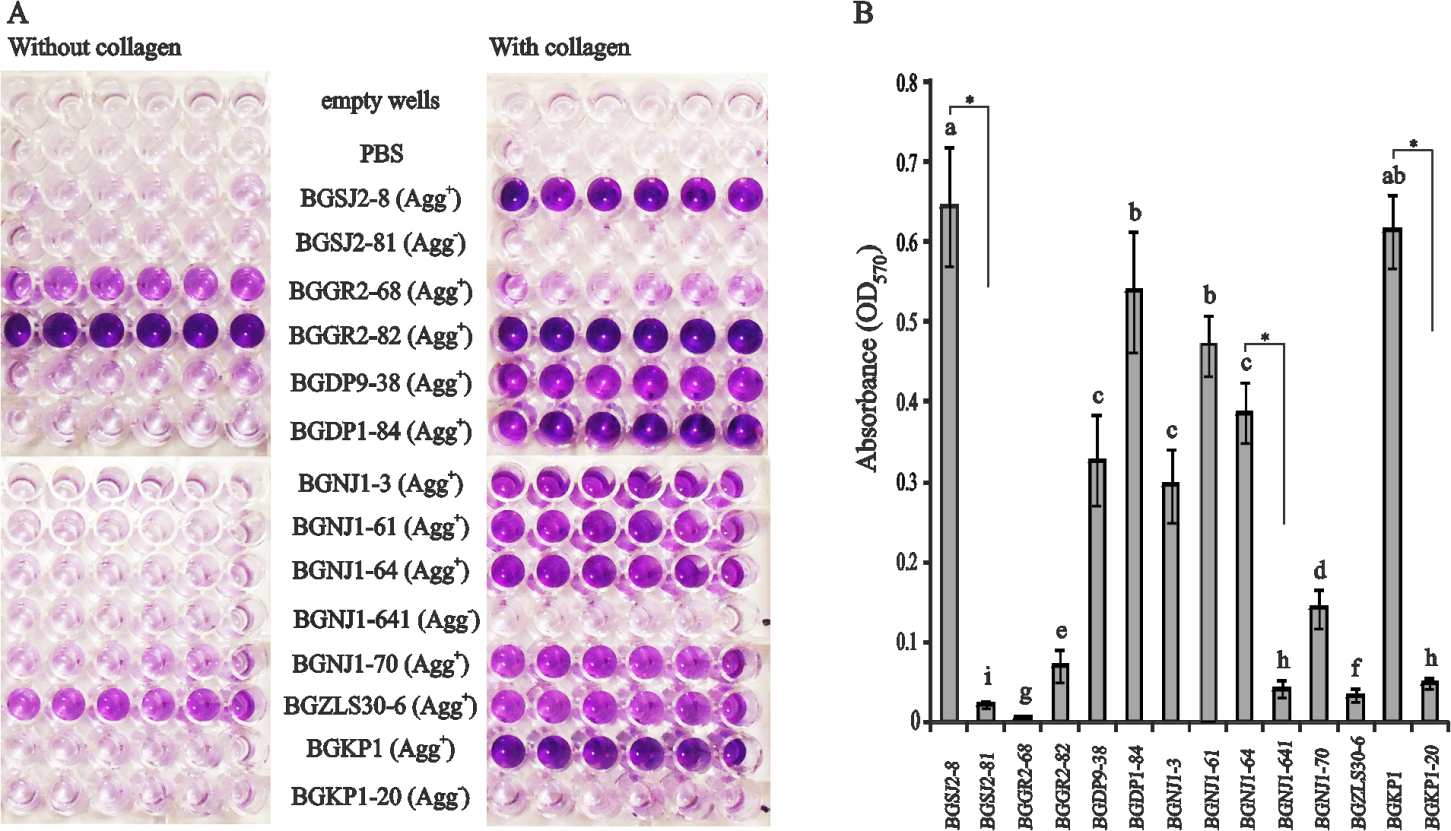
Collagen-binding assay. Binding of different *Lactobacillus* strains (including control strains) to immobilized collagen (A) and graphical presentation of results (B) expressed as normalized A_570_ values. Error bars show standard deviations. Asterisk indicates a significant difference between aggregating strains and their non-aggregating derivatives (p<0.001). Within columns, values with different superior letters differ significantly (P<0.05).

Based on the knowledge that the aggregation factor from lactococci (AggL) contains a repeat region known to mediate collagen binding, we tested the binding abilities of the selected lactobacilli (Fig 2). Cells of tested *Lactobacillus* strains adhered to immobilized collagen to different extents (Fig 2A). The most adhesive strain was BGSJ2-8 (adhesion value 0.64), while the least adhesive strain was BGNJ1-70 (adhesion value 0.14) (Fig 2B). The strains BGGR2-68, BGGR2-82, and BGZLS30-6 showed no ability to bind collagen, but these strains were able to adhere to the plastic surface of multiwell plates. Adhesion values for non-aggregating strains ranged from 0.02 to 0.05. Significant differences in adherence were obtained for aggregating strains and their non-aggregating derivatives, indicating a role of aggregation factor(s) in the specific interaction with collagen.

### Sequencing and sequence analysis of the pALb35 construct

Upon the confirmation of the auto-aggregation capacity of the strain carrying construct pALb35, the complete SacI fragment was subjected to sequencing but numerous repetitive sequences at the 3’-terminal part of the cloned DNA interfered. To ensure correct sequence determination, a combination of primer walking, subcloning and nested deletion analysis using the ExoIII/S1 treatment was applied. The 11377-bp fragment contained four ORFs (three transposase genes and one novel gene named *aggLb*) and had 42.28% G+C content. The transposase genes (two of them located upstream and one downstream of the *aggLb* gene) have already been found in other *Lactobacillus* strains sharing almost 100% identity. The novel *aggLb* gene, responsible for expression of auto-aggregation phenotype of BGNJ1-64, consists of 8994 bp. The first 4523-bp portion of *aggLb* gene shares 99% identity with genes coding for hypothetical proteins from *Lactobacillus casei* W56 (GenBank Accession No. HE970765), LC2W (GenBank Accession No. CP002617) and BDII (GenBank Accession No. CP002619). The rest of the gene, containing numerous repeats, does not show significant identity with any known gene. We identified two large direct repeats of 1196 bp inside of the gene (the first corresponding to nucleotides 3883–5078 and the second corresponding to nucleotides 5293–6488) with 100% identity. This region could be otherwise segmented in 18 repeats with almost 100% identity on nucleotide level. To confirm that the repeated sequences in the *aggLb* gene are not a result of duplications that occurred in *E*. *coli* during cloning and propagation, a hybridization experiment was performed. Plasmids from the BGNJ1-64 strain and clone pALb35 were digested with different restriction enzymes and probed with pALb35 (S3 Fig). The same fragments sizes were detected carrying the *aggLb* gene in both the natural plasmid and the clone, confirming that the 11377-bp clone matches the gene of the original strain.

### *In silico* analysis of the AggLb protein

**Fig 3:**
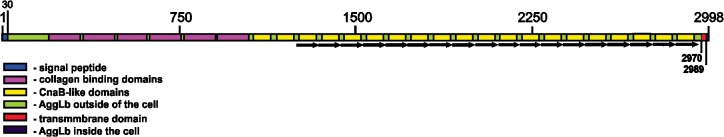
Schematic representation of AggLb protein. Boxes indicate domains of protein. Arrows indicate repeats.

A schematic representation of AggLb is shown in Fig 3. Protein sequence analysis revealed that the N-terminal half of AggLb shares 98% identity with hypothetical proteins in *Lb*. *casei* W56 (YP_007327693.1), BD-II (YP_005861272.1), and LC2W (YP_005861220.1). The C-terminal half of AggLb did not show continuous identity with any known protein. AggLb appears to be the largest cell wall-anchored protein in *Lactobacillus* (318.6 kDa) and is rich in threonine (12.5%) and lysine (10.2%). The protein contains two motifs important for cell adhesion, namely collagen-binding domains in the N-terminal portion (6 times repeated at aa locations 209–340, 361–496, 507–625, 635–757, 772–904 and 906–1047) and collagen-binding protein B (CnaB)-like domains (K[ILV]ASGLTTDAKGQI[QK]VNDLKP[GS]DYYFVETAAPAGYELNDSKLNFTVELQT) in the C-terminal region (20 times repeated at aa positions 1078–1144, 1178–1247, 1270–1340, 1364–1435, 1458–1529, 1552–1623, 1646–1717, 1740–1810, 1834–1905, 1928–1999, 2022–2093, 2116–2186, 2210–2280, 2304–2374, 2398–2468, 2492–2562, 2586–2656, 2680–2750, 2774–2844 and 2868–2932). The N-terminal cleavage site of the signal peptide is predicted to be between aa-positions 30 and 31 (http://www.cbs.dtu.dk/services/SignalP/) [36]. AggLb is predicted to possess a cell-wall anchoring motif (LPXTG) and one transmembrane domain at position 2970–2989 (http://bioinformatics.biol.uoa.gr/CW-PRED/) [37]. In addition, AggLb has 18 almost identical consecutive repeats in the C-terminal region, each composed of 94 aa (GSVVLNKTDSDTGKALSGAVFDLYKKDGTKIASGLMTDAKGQIKVNDLKPGDYYFVETAAPAGYELNDSKLNFTVELQTTAKVATVSATNAEKT) starting at position 1255 and ending at position 2946 aa without spaces between repeats. The first and last repeats show the highest difference in aa composition.

### Heterologous expression of the *aggLb* gene

The construct pALb35 successfully restored the aggregation phenotype in heterologous hosts *L*. *lactis* BGKP1-20 and *E*. *faecalis* BGZLS10-27 after transformation. This result supports the conclusion that *aggLb* gene is sufficient for the expression of the aggregation phenotype in closely related species. It should be noted that the aggregation ability of enterococcal cells carrying the *aggLb* gene differed from the levels obtained in *Lactococcus* and *Lactobacillus* in the type and size of cell aggregates.

### The role of *aggLb* in collagen binding

**Fig 4:**
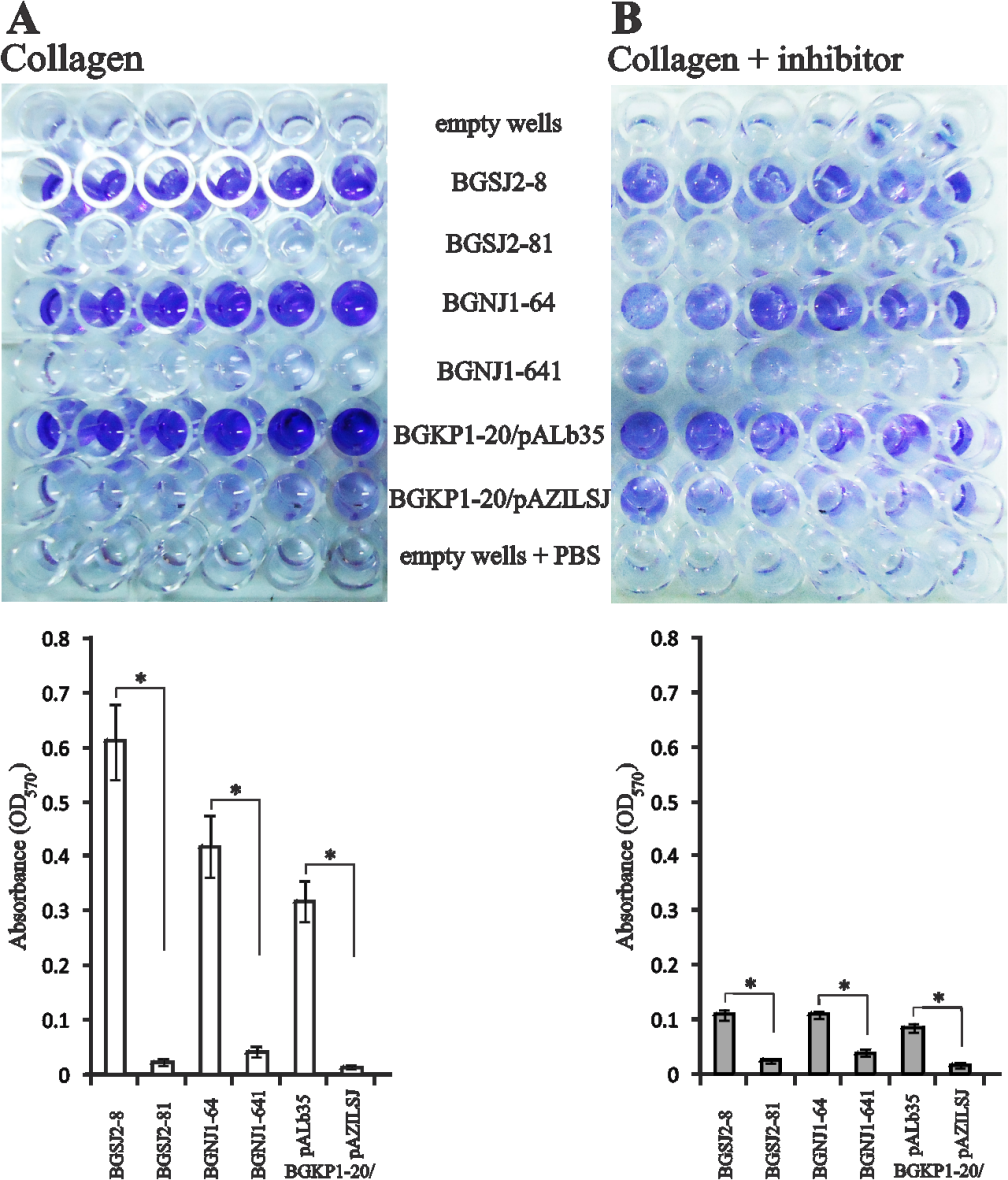
Collagen-binding and inhibition assays. Binding (A) and inhibition of binding by heparin sodium salt (B) to immobilized collagen of selected natural Agg+ isolates and strains with heterologously expressed AggLb (including control strains). Lower panels show graphical presentation of results expressed as normalized A_570_ values. Error bars show standard deviations. Asterisk indicates a significant difference (p<0.001) between aggregating strains and their non-aggregating derivatives, as well as for aggregating strains untreated and treated with the inhibitor.

The results obtained for Agg^+^ strains and their non-aggregating, plasmid cured derivatives, indicate a strong correlation between aggregation ability and collagen binding. To prove and eventually quantify the contribution of the *aggLb* gene (responsible for auto-aggregation) to collagen binding, selected *Lactobacillus* strains (BGSJ2-8 and BGNJ1-64), their non-aggregating derivatives (BGSJ2-81 and BGNJ1-641), and heterologous hosts (BGKP1-20/pALb35, expressing *aggLb*, and BGKP1-20/pAZILSJ, the corresponding non-aggregating control) were tested. Strains BGSJ2-8, BGNJ1-64, and BGKP1-20/pALb35 showed significantly higher degrees of adhesion (> 0.3) to immobilized collagen compared to corresponding non-aggregating derivatives (Fig 4A). Because the contribution of heterologously expressed *aggLb* to collagen binding was even higher than differences between WT strains and their plasmid cured derivatives, overall binding of bacterial cells is possibly dose-dependent on AggLb.

Heparin sodium salt and other sulphated glycosaminoglycans strongly inhibit binding of bacterial cells to ECM proteins. To measure the specificity of bacterial collagen binding, the strains listed above were subjected to the collagen-binding assay in the presence of heparin sodium salt (Fig 4B). It was found that heparin sodium salt significantly reduced collagen binding, confirming that the specific interaction of tested bacteria with collagen is mediated by the large cell-surface protein AggLb.

### Hydrophobicity of selected Agg^+^ strains and their derivatives

**Fig 5:**
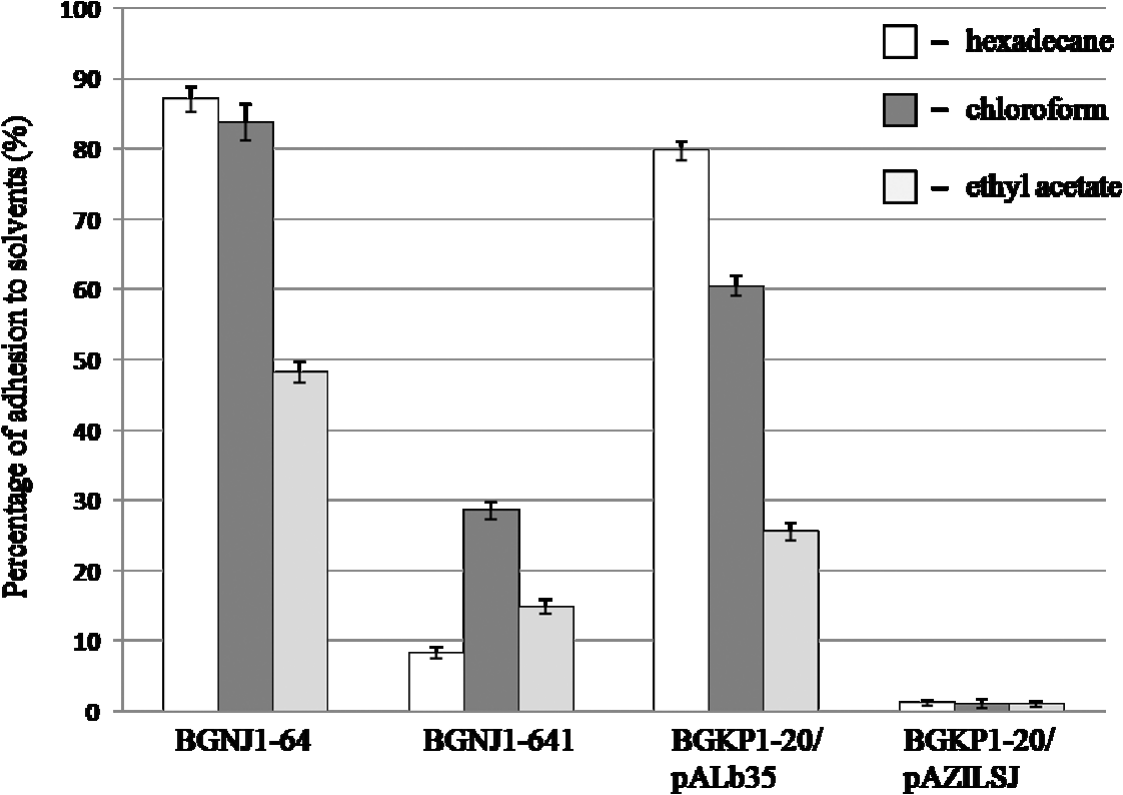
Microbial adhesion to solvents. The percent adhesion of selected strains to different solvents was determined by the MATS assay. The values are the mean of three independent experiments and error bars represent standard deviations.

To analyze whether cell-surface characteristics are dependent on aggregation factors, the hydrophobicity MATS assay was performed with BGNJ1-64 and BGKP1-20/pALb35. The BGNJ1-641 and BGKP1-20/pAZILSJ derivatives were used as controls. The three solvents used in this study were hexadecane, an apolar solvent, chloroform, a monopolar and acidic solvent and ethyl acetate, a monopolar and basic solvent (Fig 5). Only microbial adhesion to hexadecane reflects cell-surface hydrophobicity or hydrophilicity because electrostatic interactions are absent. The values of MATS obtained with chloroform and ethyl acetate were regarded as a measure of electron-donor/basic and electron-acceptor/acidic characteristics, respectively, of the bacteria. All three solvents have similar van der Waals properties.

Analyzed aggregation-positive strains exhibited very high percentages of adhesion to hexadecane. The difference in hydrophobicity between BGNJ1-64 and its non-aggregating derivative BGNJ1-641 (78.73%) was significant and remarkable, along with the difference between BGKP1-20/pALb35 and its non-aggregating derivative BGKP1-20/pAZILSJ (78.59%). Surface characteristics of the natural isolate BGNJ1-64 and the derivative with expressed AggLb (BGKP1-20/pALb35) were very similar, strongly indicating that the contribution of AggLb to the hydrophobicity of the carrier strains is almost 100%. Additionally, both strains with aggregation ability demonstrated higher affinities for chloroform (an acidic solvent) and a lower percentage of adhesion to ethyl acetate (a basic solvent). These results indicate that AggLb is able to provide strong electron-donor and weaker electron-acceptor features to the bacteria, confirmed by their hydrophilic cell-surface properties. Moreover, results of the MATS test showed no significant difference between the BGNJ1-64 and KP1-20/pALb35 strains regarding adhesion to the same solvent, thus confirming crucial role of AggLb in the determination of cell-surface properties.

### Exclusion of *S*. *aureus* ATCC25923 and *E*. *coli* O157:H7 by selected Agg^+^ strains and their derivatives

Because aggregation-positive strains, which carry the *aggLb* gene, demonstrated strong collagen binding affinity, the ability of AggLb to decrease pathogen adhesion to collagen was tested. Results obtained for pathogen exclusion by lactobacilli are shown in Table 2. The pathogenic strain**s** *S*. *aureus* ATCC25923 and *E*. *coli* O157:H7 showed strong binding to immobilized collagen (466 ± 23.43; 420.6 ± 19.36 CFU/well, respectively). When immobilized collagen was first incubated with *Lactobacillus* strains, adhesion of the pathogens to collagen was significantly inhibited. Remarkable differences in the inhibition of the adhesion ability of *S*. *aureus* ATCC25923 (80.83%) and *E*. *coli* O157:H7 (55.39%) were observed between BGNJ1-64 and its non-aggregating derivative BGNJ1-641. The differences between BGSJ2-8 and its non-aggregation derivative BGSJ2-81 were 57.22% (for *S*. *aureus* ATCC25923) and 69.75% (for *E*. *coli* O157:H7). Additionally, to determine contribution of *aggLb* to the inhibition of *S*. *aureus* ATCC25923 and *E*. *coli* O157:H7 adhesion to collagen, the differences in inhibition of adhesion ability between aggregating strains BGKP1 and BGKP1-20/pALb35 compared to non-aggregating derivative BGKP1-20/pAZILSJ were analyzed. The quantitative binding of *S*. *aureus* ATCC25923 decreased from 337.33 ± 17.0 CFU/well (coated with BGKP1-20/pAZILSJ) to 68.0 ± 6.24 and 44.33 ± 7.23 CFU/well (coated with BGKP1 and BGKP1-20/pALb35, respectively). Similarly, the binding of *E*. *coli* O157:H7 to collagen decreased from 275.0 ± 20.31 CFU/well (coated with BGKP1-20/pAZILSJ) to 38.0 ± 7.31 and 35.2 ± 4.15 CFU/well (coated with BGKP1 and BGKP1-20/pALb35, respectively). These comparative assessments demonstrate that BGKP1 (carrier of *aggL* gene) inhibits adhesion of *S*. *aureus* ATCC25923 for 57.79% and *E*. *coli* O157:H7 for 56.35% on immobilized collagen, while BGKP1-20/pALb35 strain (carrier of *aggLb* gene) inhibits adhesion of *S*. *aureus* ATCC25923 for 62.87% and *E*. *coli* O157:H7 for 57.01% on the same matrix. Our results highlight the role of AggLb in the inhibition of pathogens adhesion in addition to the specific binding to collagen.

**Table 2:** Exclusion of *S*. *aureus* ATCC25923 and *E*. *coli* O157:H7 by selected *Lactobacillus* strains.

Experimental setting	Adhered cells of *S*. *aureus* (CFU/well)	Adhered cells of *E*. *coli* O157:H7 (CFU/well)
Immobilized collagen (control)	466 ± 23.43	420.6 ± 19.36
Immobilized collagen coated with:		
*Lactobacillus paracasei* subsp. *paracasei*		
BGSJ2-8^+^	47.33 ± 5.51	68.4 ± 6.69
BGSJ2-81^-^	314.0 ± 15.10	361.8 ± 16.79
BGNJ1-64^+^	41.0 ± 4.58	34.4 ± 5.03
BGNJ1-641^-^	417.66 ± 25.38	267.4 ± 18.51
*Lactococcus lactis* subsp. *lactis*		
BGKP1^+^	68.0 ± 6.24	38.0 ± 7.31
BGKP1-20/pAZILSJ^-^	337.33 ± 17.0	275.0± 20.31
BGKP1-20/pALb35^+^	44.33 ± 7.23	35.2 ± 4.15

^+^- aggregation-positive phenotype;

^-^- aggregation-negative phenotype

### Screening selected Agg^+^ strains for the presence of *aggLb*

Because we were able to detect *Lactobacillus* strains with different aggregation characteristics, detection of other genes responsible for aggregation in the selected lactobacilli was performed by PCR and Southern blot.

**Fig 6:**
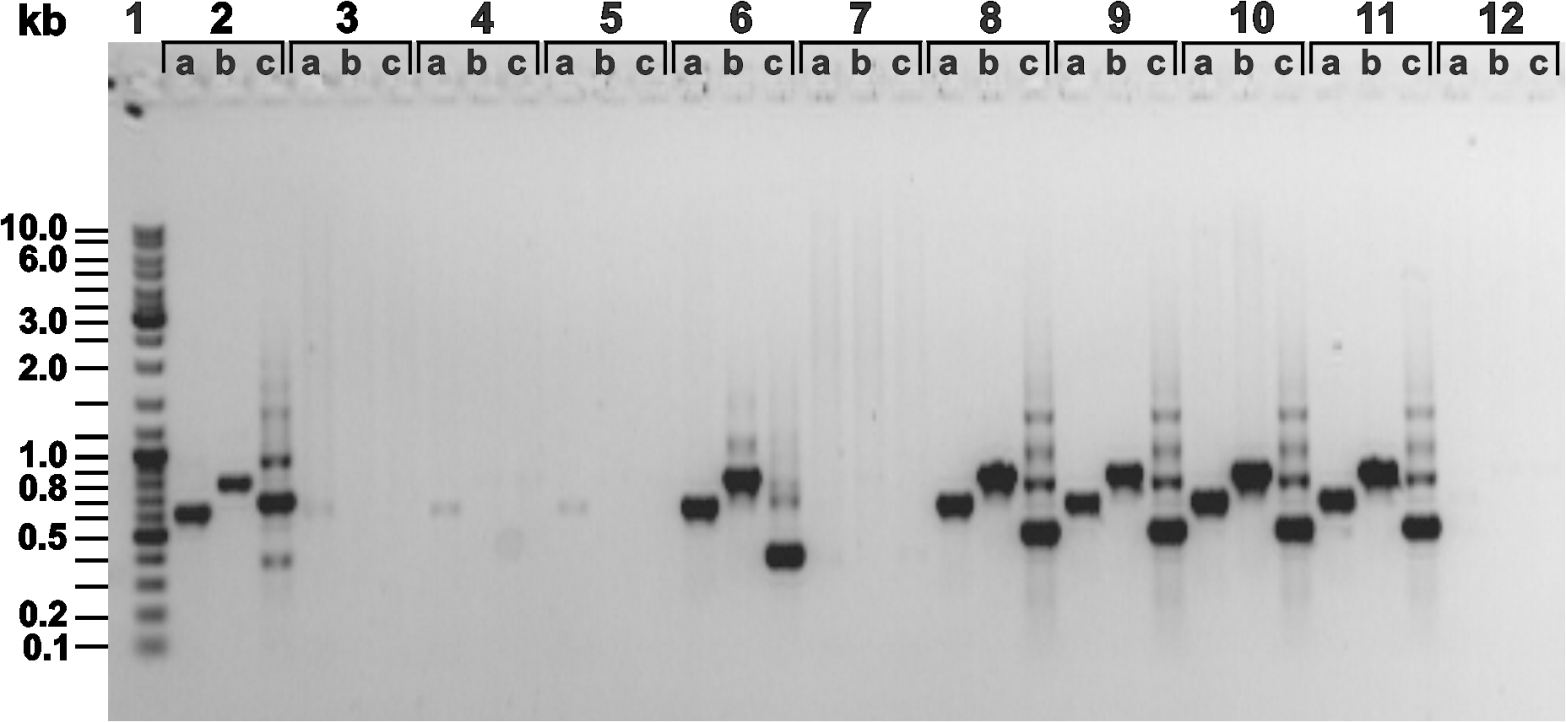
Screening of selected Agg^+^ strains for presence of *aggLb* gene by PCR. Agarose gel electrophoresis of *aggLb* gene fragments obtained by PCR amplification with different primers (Agg1 Fw—Agg1 Rev; lanes a), (Agg2 Fw—Agg2 Rev; lanes b), (Agg3 Fw—Agg3 Rev; lanes c). Lanes: 1- GeneRuler 1 kb Plus DNA Ladder; 2- BGSJ2-8; 3- BGAR75; 4- BGGR2-68; 5- BGGR2-82; 6- BGDP1-84; 7- BGDP9-38; 8- BGNJ1-3; 9- BGNJ1-61; 10- BGNJ1-64; 11- BGNJ1-70; 12-BGZLS30-6.

Specific sets of primers (Agg1Fw—Agg1Rev, Agg2Fw—Agg2Rev, Agg3Fw—Agg3Rev, S1 Table) were designed for amplification of *aggLb* from the total and plasmid DNA isolated from selected lactobacilli strains. PCR fragments obtained from five (BGSJ2-8, BGDP1-84, BGNJ1-3, BGNJ1-61, BGNJ1-70) of 10 strains (Fig 6) were sequenced. The *aggLb* genes of these five aggregating lactobacilli strains were highly similar (99% identity on the nucleotide level) to that of BGNJ1-64, although the numbers of repeats were not determined.

In addition, a hybridization experiment with *aggLb* as a probe was performed with digested total and plasmid DNA of all selected lactobacilli strains. The results of hybridization fully support those obtained by PCR. The five strains in which *aggLb* was amplified by PCR showed similar restriction enzyme profiles, while the other five strains did not give any hybridization signal.

## Discussion

The main goal of this study was to investigate possible role of *Lactobacillus* aggregation factors in determination of features favored in probiotics, such as collagen binding and protection from adhesion of pathogenic bacteria. The ability of pathogenic bacteria to adhere to distinct components of the ECM, such as collagen and fibronectin, is enabled by the expression of ECM-binding proteins, termed adhesins. Adhesins are important virulence factors of pathogens, as they are involved in the initiation of infection [38]. Some lactobacilli represent beneficial microflora (probiotics) inhabiting the GI and UG tracts of humans and animals. The ability of probiotic lactobacilli to form cellular aggregates is considered desirable because this feature potentially inhibits adherence of pathogenic bacteria to intestinal mucosa. This inhibition may be accomplished by formation of a barrier via auto-aggregation on the intestinal mucosa and preventing colonization of pathogens through competition for the same targets or, alternatively, probiotics may directly co-aggregate with pathogenic bacteria and facilitate their clearance [11, 39, 40]. Considering the importance of aggregation phenomena for human health, the laboratory collection was screened for *Lactobacillus* strains exhibiting aggregation ability for further investigation. It was found that aggregation ability is a rare phenotype among lactobacilli (1–2%). Bearing in mind the complexity of the mechanisms and components involved in cell aggregation and adherence, the species specificity, environmental dependence, and contribution to multiple probiotic functional roles [11, 15, 16, 17, 21, 41], we decided to analyze the aggregation-promoting factor specific for *Lb*. *paracasei* and its role in collagen binding and pathogen exclusion. We attempted to determine whether the presence and expression of the aggregation-promoting factor is correlated with collagen binding by *Lactobacillus* strains.

It is interesting that we were able to localize genes for aggregation on large plasmids in only two of the selected strains. A novel shuttle cloning vector pAZILSJ was constructed to clone the *aggLb* gene of *Lb*. *paracasei* subsp. *paracasei* BGNJ1-64 and study its expression in heterologous *Lactococcus* and *Enterococcus* strains. AggLb belongs to a group of collagen-binding proteins, similar to an aggregation-promoting factor recently discovered in lactococci [21] and biosurfactants produced by several *Lactobacillus* species [42]. To our knowledge, the 318.6-kDa AggLb is the largest aggregation-promoting protein discovered so far in LAB. It has a significantly higher molecular weight than lactococci AggL (193.4 kDa). Both proteins have similar architecture, although they have no similarity at the aa level. AggLb is much longer due to the presence of 20 CnaB-like domains, in contrast to the lactococcal protein, which bears only 7 CnaB-like domains. *In silico* domain prediction by MEME (http://meme.nbcr.net) [43] suggests the involvement of CnaB-like domains in cell aggregation. Similar to AggLb, which has 18 repeats of 94 aa, biofilm-associated proteins have 13 identical 258-nucleotide tandem repeats encoding reiterations of an 86-aa sequence in the C-terminal region [44]. Both genes (*aggLb* and *aggL*) are located on the plasmids, which enable the potential transfer of these factors among the microbial population. Additionally, the fact that bacteria maintain and procure genes coding for aggregation factors without direct selection, despite the energy cost of plasmid replication and gene expression, suggests that this feature provides a benefit for the cell.

Analysis of the primary structure of AggLb revealed a domain organization similar to the LPXTG-type of proteins present in Gram-positive bacteria. The LPXTG motif, a highly conserved region of the C-terminal sorting signal, plays a crucial role in covalent linkage of many cell-wall-associated surface proteins to the nascent pentaglycine cross-bridge of peptidoglycan [45]. The CnaB-like domain is the most abundant domain of AggLb and is likely involved in mediation of bacterial adherence to collagen. In addition, repeated units have been suggested to serve as a ‘stalk’ that exposes the region crucial for adherence to the bacterial surface, thus facilitating bacterial adherence to collagen [46]. Finally, heterologous expression of *aggLb* revealed the indispensable role of AggLb in aggregation phenomena and in the strong and specific binding to collagen. The collagen-binding ability of aggregation-positive strains expressing AggLb may be beneficial characteristic for probiotic strains because it could provide protection of the EMC from adhesion of pathogens, most likely by competition for the same attachment sites. Similar results were obtained by other authors for different surface proteins. Removal of the S-layer proteins from *Lb*. *crispatus* ZJ001 reduced auto-aggregation and adhesion of *Salmonella typhimurium* and *E*. *coli* O157:H7 to HeLa cells via competitive exclusion [47]. The protective effect of *Lb*. *reuteri* on inhibition of adherence of *S*. *aureus* to keratinocytes was mediated by competitive exclusion. Keratinocyte survival was significantly higher when the probiotic was applied prior to or simultaneously with *S*. *aureus* infection, but not when it was added after infection had commenced [48]. The number of adhering *S*. *aureus* and *S*. *epidermidis* cells after co-incubation with biosurfactants from *Lb*. *acidophilus* was reduced by 5–56% in a strain- and dose-dependent manner [49]. Zarate *et al*. demonstrated the ability of *Lb*. *paracasei* CRL1289, a human vaginal strain with probiotic properties, to prevent the vaginal colonization by an uropathogenic strain of *S*. *aureus* [50].

Based on the presented results we conclude that AggLb is a novel aggregation-promoting factor from *Lb*. *paracasei* subsp. *paracasei* BGNJ1-64 that contributes to the diverse functions and behavior of the carriers, including strong aggregation ability, strong and specific interaction with collagen through changes to cell-surface properties. AggLb is most likely involved in protection of the host ECM from pathogen infection by a mechanism of competitive exclusion. Based on the morphological characteristics and the degree of aggregation in heterologous strains used in this study, it can be speculated that even though AggLb is crucial for aggregation, some additional host factors may have a modulatory effect on the aggregation phenotype.

We found at least two types of aggregation-promoting factors in *Lb*. *paracasei*. We cloned and characterized one of these factors, AggLb, which was present in more than 50% of the analyzed strains. The other(s) remains uncharacterized and will be addressed in future work. Our results, together with other published data, indicate the presence of diverse aggregation factors in lactobacilli, even within the same species. Because the interest in the aggregation of lactobacilli is related to their probiotic function [33], further experiments will be primarily focused on the interaction of AggLb with human epithelial cells and the role of the interaction in the adhesion and possible adjustment of the immune response. Moreover, it will be interesting to study contribution of the different domains and repeats of AggLb to the modulation of the aggregation phenotype.

## Supporting Information

S1 Fig Visual aggregation assay. Aggregation ability of *Lb*. *paracasei* subsp. *paracasei* strains in liquid growth medium after overnight cultivation (A) and vigorous mixing (B). Lanes: 1- growth medium; 2- BGSJ2-8; 3- BGGR2-68; 4- BGGR2-82; 5- BGDP1-84; 6- BGDP9-38; 7- BGNJ1-3; 8- BGNJ1-61; 9- BGNJ1-64; 10- BGNJ1-70; 11- BGZLS30-6; 12- BGAR75.
(TIF)

S2 Fig Plasmid profiles of selected *Lb*. *paracasei* subsp. *paracasei* strains expressing aggregation ability. Lanes: 1- GeneRuler 1 kb Plus DNA Ladder; 2- BGSJ2-8; 3- BGGR2-68; 4- BGGR2-82; 5- BGDP1-84; 6- BGDP9-38; 7- BGNJ1-3; 8- BGNJ1-61; 9- BGNJ1-64; 10- BGNJ1-70; 11- BGZLS30-6; 12- BGAR75.
(TIF)

S3 Fig Southern blot hybridization experiment with clone pALb35 as a probe. (A) Agarose gel with total plasmid DNA isolated from *Lb*. *paracasei* subsp. *paracasei* BGNJ1-64 (lanes 2, 4, 6, 8, 10 and 12) and clone pALb35 (lanes 3, 5, 7, 9, 11 and 13), digested with SacI (lanes 2 and 3), XbaI (lanes 4 and 5), EcoRV (lanes 6 and 7), EcoRI (lanes 8 and 9), PstI (lanes 10 and 11), and SphI (lanes 12 and 13), (B) Membrane after hybridization of the digested DNA with the probe (lane 14, probe as positive control). Lanes 1 and 15, GeneRuler 1 kb Plus DNA Ladder.
(TIF)

S1 Table Primers used in the study. (DOCX)

S2 Table Auto-aggregation abilities of selected lactobacilli determined by spectrophotometry measurements (OD 600) within 5h. (DOCX)
