# Supplementary figures and images for: AggLb Is the Largest Cell-Aggregation Factor from Lactobacillus paracasei Subsp. paracasei BGNJ1-64, Functions in Collagen Adhesion, and Pathogen Exclusion In Vitro
Source: PLoS One. 2015 May 8;10(5):e0126387. doi: 10.1371/journal.pone.0126387 (PMC4425601; doi:10.1371/journal.pone.0126387)

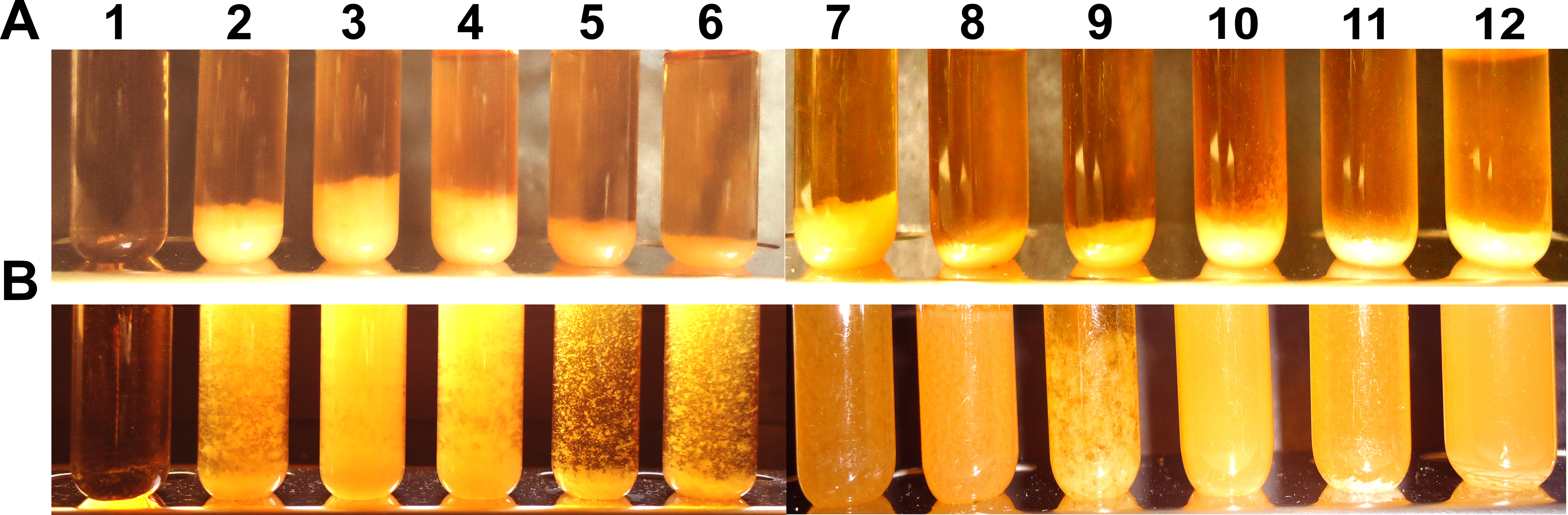

Supplement: S1 Fig — Aggregation ability of Lb. paracasei subsp. paracasei strains in liquid growth medium after overnight cultivation (A) and vigorous mixing (B). Lanes: 1- growth medium; 2- BGSJ2-8; 3- BGGR2-68; 4- BGGR2-82; 5- BGDP1-84; 6- BGDP9-38; 7- BGNJ1-3; 8- BGNJ1-61; 9- BGNJ1-64; 10- BGNJ1-70; 11- BGZLS30-6; 12- BGAR75. (TIF) [file pone.0126387.s001.tif]

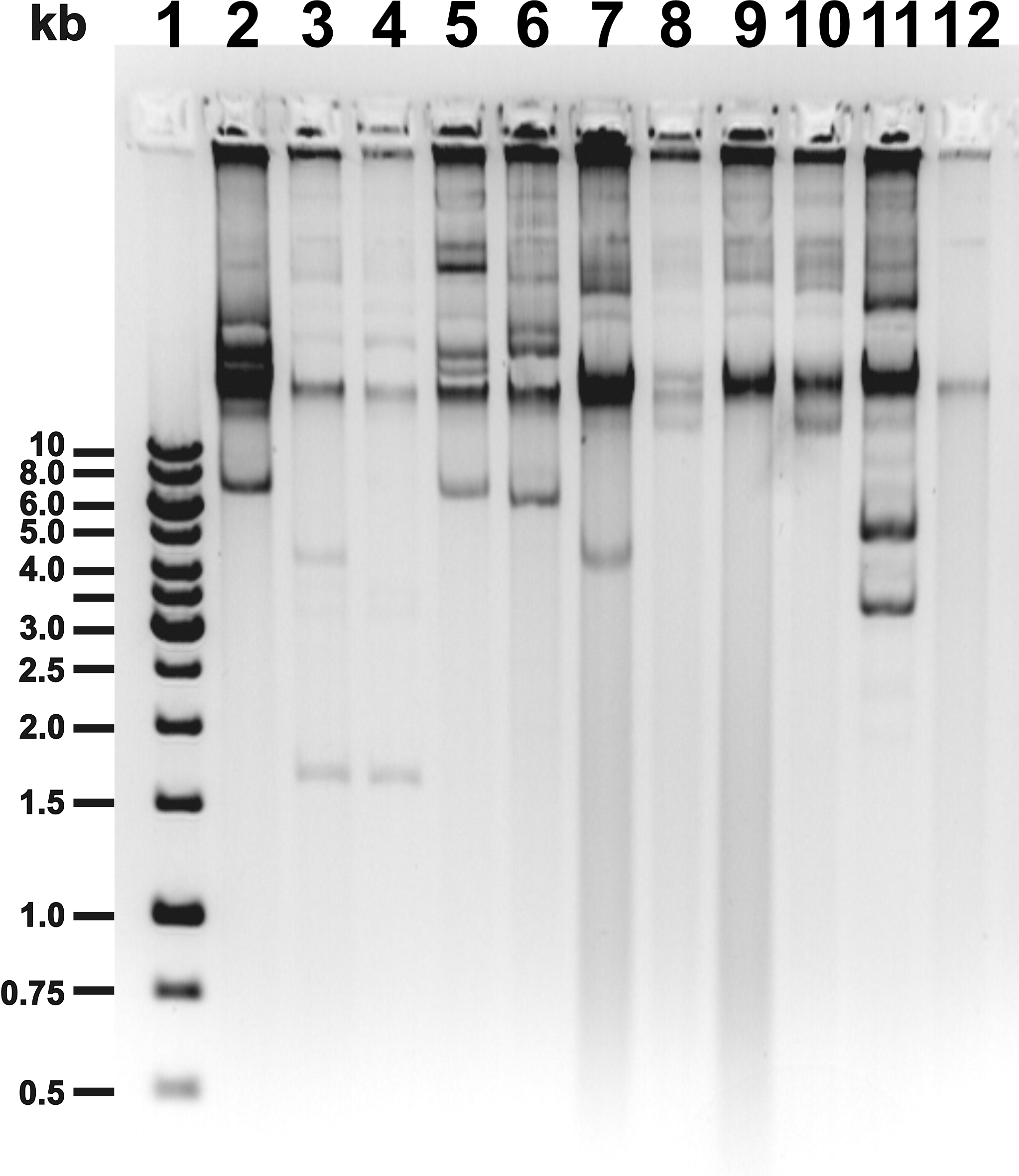

Supplement: S2 Fig — Lanes: 1- GeneRuler 1 kb Plus DNA Ladder; 2- BGSJ2-8; 3- BGGR2-68; 4- BGGR2-82; 5- BGDP1-84; 6- BGDP9-38; 7- BGNJ1-3; 8- BGNJ1-61; 9- BGNJ1-64; 10- BGNJ1-70; 11- BGZLS30-6; 12- BGAR75. (TIF) [file pone.0126387.s002.tif]

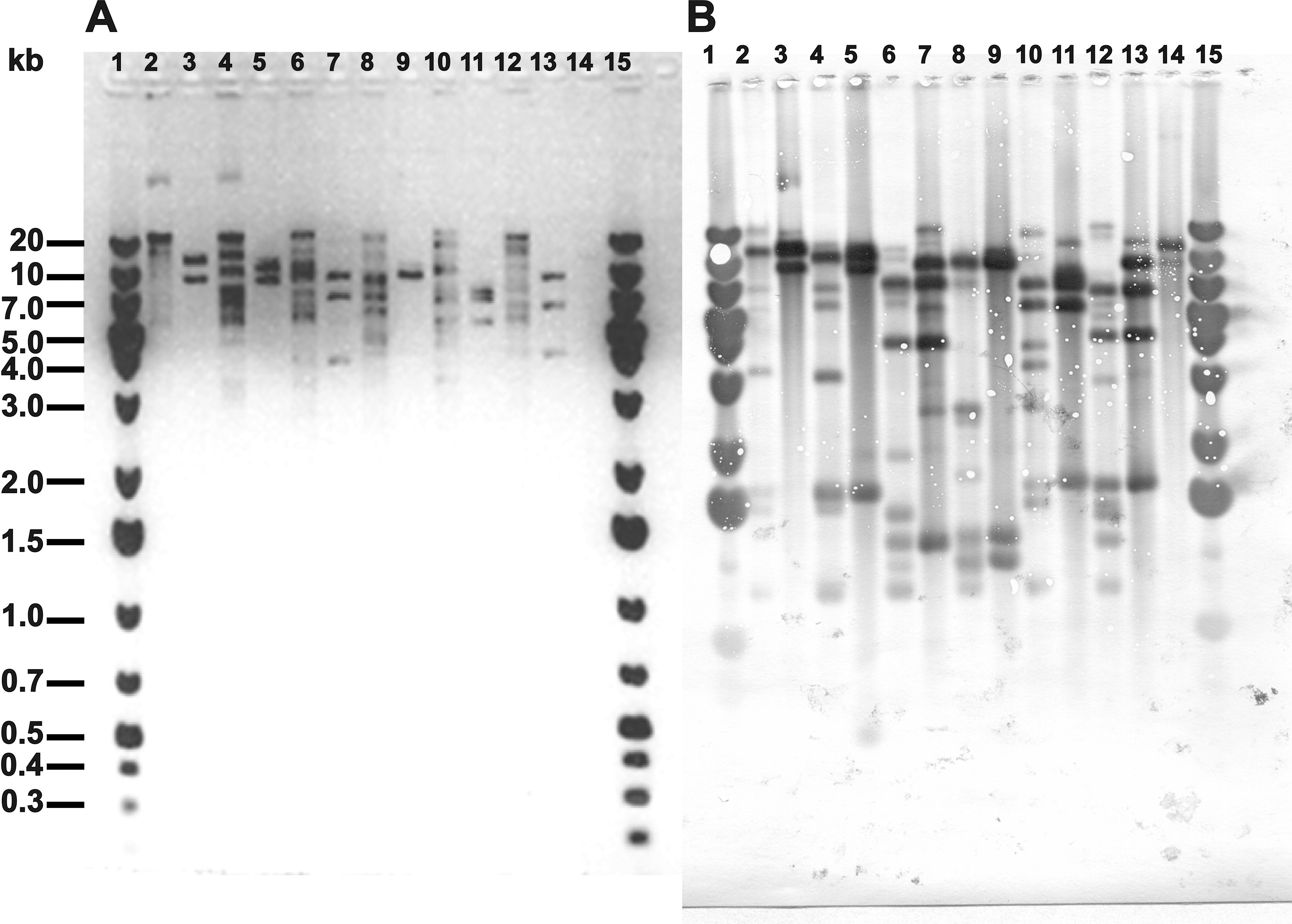

Supplement: S3 Fig — (A) Agarose gel with total plasmid DNA isolated from Lb. paracasei subsp. paracasei BGNJ1-64 (lanes 2, 4, 6, 8, 10 and 12) and clone pALb35 (lanes 3, 5, 7, 9, 11 and 13), digested with SacI (lanes 2 and 3), XbaI (lanes 4 and 5), EcoRV (lanes 6 and 7), EcoRI (lanes 8 and 9), PstI (lanes 10 and 11), and SphI (lanes 12 and 13), (B) Membrane after hybridization of the digested DNA with the probe (lane 14, probe as positive control). Lanes 1 and 15, GeneRuler 1 kb Plus DNA Ladder. (TIF) [file pone.0126387.s003.tif]
