# Supplementary material for: AggLb Is the Largest Cell-Aggregation Factor from Lactobacillus paracasei Subsp. paracasei BGNJ1-64, Functions in Collagen Adhesion, and Pathogen Exclusion In Vitro
Source: PLoS One. 2015 May 8;10(5):e0126387. doi: 10.1371/journal.pone.0126387 (PMC4425601; doi:10.1371/journal.pone.0126387)
Supplement: S1 Table — (DOCX) [file pone.0126387.s004.docx]

**S1 Table. Primers used in the study.**

| Primers | Primer sequence | Amplicon |
| --- | --- | --- |
| - Agg1 Fw   Agg1 Rev | - 5’-TTGTAGGAGCCGTTACATCT-3’   5’-TTGTGTGCGATCAACAGTAT-3’ | 81-688 bp of *aggLb* gene^a^ |
| Agg2 Fw  Agg2 Rev | 5’-CATATCCTTCGCTAATGCTT-3’  5’-CTTCCCTGGATAGATCACAA-3’ | 2113-2887 bp of *aggLb* gene^a^ |
| Agg3 Fw  Agg3 Rev | 5’-GCTGAACTTCACGGTTG-3’  5’-AGAACCGAAAGTATC-3’ | 8484-8951 bp of *aggLb* gene^a^ |
| BclIF  BclIR | 5'-GTTTTTGATCATGTATGGGG-3'  5' -CCGCGCTGATCATCGTTGGTG-3' | Ori region and *repB1* gene^b^ |

^a^*Lb. paracasei* subsp. *paracasei* BGNJ1-64, *aggLb* gene, Acc. No. HG008907.2

^b^*Lb. paracasei* subsp. *paracasei* BGSJ2-8, plasmid pSJ2-8, Acc. No. NC_012222
