# Supplementary material for: AggLb Is the Largest Cell-Aggregation Factor from Lactobacillus paracasei Subsp. paracasei BGNJ1-64, Functions in Collagen Adhesion, and Pathogen Exclusion In Vitro
Source: PLoS One. 2015 May 8;10(5):e0126387. doi: 10.1371/journal.pone.0126387 (PMC4425601; doi:10.1371/journal.pone.0126387)
Supplement: S2 Table — (DOCX) [file pone.0126387.s005.docx]

**S2 Table. Auto-aggregation abilities of selected lactobacilli determined by spectrophotometry measurements (OD 600) within 5h.**

| Strain | Auto-aggregation (%) | | | | |
| --- | --- | --- | --- | --- | --- |
|  | 1h | 2h | 3h | 4h | 5h |
| *Lactobacillus paracasei* subsp. *paracasei* |  |  |  |  |  |
| BGSJ2-8^+^ | 41.62 ± 0.71 | 48.12 ± 0.85 | 51.51 ± 0.42 | 56.84 ± 1.12 | 59.81 ± 0.63 |
| BGGR2-68^+^ | 45.23 ± 0.93 | 54.22 ± 0.09 | 63.39 ± 1.93 | 69.59 ± 0.40 | 72.99 ± 0.64 |
| BGGR2-82^+^ | 13.52 ± 0.74 | 16.09 ± 1.94 | 23.48 ± 2.43 | 35.26 ± 1.38 | 38.26 ± 0.43 |
| BGDP1-84^+^ | 41.43 ± 1.29 | 49.14 ± 1.06 | 52.87 ± 0.85 | 56.76 ± 1.95 | 58.81 ± 1.69 |
| BGDP9-38^+^ | 14.63 ± 0.33 | 25.28 ± 1.14 | 37.85 ± 1.84 | 44.27 ± 1.01 | 50.57 ± 1.64 |
| BGNJ1-3^+^ | 20.43 ± 0.62 | 27.48 ± 0.98 | 37.16 ± 1.37 | 43.65 ± 1.57 | 48.33 ± 0.63 |
| BGNJ1-61^+^ | 44.77 ± 1.76 | 46.25 ± 1.11 | 62.19 ± 2.01 | 67.62 ± 1.43 | 73.58 ± 0.79 |
| BGNJ1-64^+^ | 40.14 ± 1.85 | 46.38 ± 1.52 | 52.87 ± 0.42 | 58.41 ± 1.75 | 62.98 ± 0.59 |
| BGNJ1-641^­-^ | 5.86 ± 0.65 | 7.09 ± 1.56 | 14.52 ± 1.42 | 18.94 ± 1.97 | 24.65 ± 1.02 |
| BGNJ1-70^+^ | 66.04 ±.69 | 69.61 ± 1.60 | 74.72 ± 1.31 | 75.65 ± 0.91 | 81.34 ± 1.34 |
| BGZLS30-6^+^ | 20.39 ± 1.86 | 28.26 ± 1.38 | 34.27 ± 2.15 | 40.28 ± 0.72 | 45.80 ± 1.81 |

^+^**-** aggregation positive phenotype; ^−^**-** aggregation negative phenotype.
